# Postmortem Alteration of Purine Metabolism in Coronary Artery Disease

**DOI:** 10.3390/metabo13111135

**Published:** 2023-11-08

**Authors:** Phakchira Somtua, Churdsak Jaikang, Giatgong Konguthaithip, Kanicnan Intui, Somlada Watcharakhom, Timothy E. O’Brien, Yutti Amornlertwatana

**Affiliations:** 1 Department of Forensic Medicine, Faculty of Medicine, Chiang Mai University, Chiang Mai 50200, Thailand; phakchira.som@cmu.ac.th (P.S.); churdsak.j@cmu.ac.th (C.J.); giatgong_k@cmu.ac.th (G.K.); kanicnan.i@cmu.ac.th (K.I.); somlada.wat@cmu.ac.th (S.W.); 2 Metabolomic Research Group for Forensic Medicine and Toxicology, Department of Forensic Medicine, Faculty of Medicine, Chiang Mai University, Chiang Mai 50200, Thailand; 3 Department of Mathematics and Statistics, Loyola University Chicago, 1032 W. Sheridan Road, Chicago, IL 60660, USA; tobrie1@luc.edu

**Keywords:** coronary artery disease, postmortem, purine pathway, xanthine, postmortem metabolism

## Abstract

A new approach for assisting in the diagnosis of coronary artery disease (CAD) as a cause of death is essential in cases where complete autopsy examinations are not feasible. The purine pathway has been associated with CAD patients, but the understanding of this pathway in postmortem changes needs to be explored. This study investigated the levels of blood purine metabolites in CAD after death. Heart blood samples (*n* = 60) were collected and divided into CAD (*n* = 23) and control groups (*n* = 37). Purine metabolites were measured via proton nuclear magnetic resonance. Guanosine triphosphate (GTP), nicotinamide adenine dinucleotide (NAD), and xanthine levels significantly decreased (*p* < 0.05); conversely, adenine and deoxyribose 5-phosphate levels significantly increased (*p* < 0.05) in the CAD group compared to the control group. Decreasing xanthine levels may serve as a marker for predicting the cause of death in CAD (AUC = 0.7). Our findings suggest that the purine pathway was interrupted by physiological processes after death, causing the metabolism of the deceased to differ from that of the living. Additionally, xanthine levels should be studied further to better understand their relationship with CAD and used as a biomarker for CAD diagnosis under decomposition and skeletonization settings.

## 1. Introduction

Cardiovascular diseases (CVDs) have continued to be the leading cause of death in Thailand and worldwide [1,2]. Approximately 19.1 mortalities per year were attributed to CVDs, and the age-adjusted death rate per 100,000 population stood at 239.8 [3] in 2020. Poor dietary habits, tobacco use, hypertension, and dyslipidemia are potential contributing factors for the development of CVDs [4,5]. Coronary artery disease (CAD) frequently progresses to atherosclerotic plaque formation, heightening susceptibility to myocardial infarctions and sudden cardiac death. Forensic investigation plays a crucial role in understanding the causes of death and contributing factors behind sudden and unexpected deaths, including cardiovascular diseases [6]. Sudden unexpected natural death (SUND) was mainly diagnosed as a cause of death by autopsy findings and pathological methods [7,8], but some of the cases cannot be involved in the performance of autopsies due to religious beliefs [9] and cultural issues.

There have been numerous attempts to identify reliable markers of coronary artery disease using molecular techniques. Troponin-T, troponin-I, and high-sensitivity troponin-T [10] are the most accurate markers in cardiovascular patients. Postmortem research has shown that troponin-T is not a specific marker for acute myocardial injury [11]. There are no specific markers in the postmortem interval effect [12]. In some studies, high-sensitivity troponin-T and tryptophan metabolites are still considered reliable diagnostic tests for sudden cardiac death [13,14].

Purines are essential molecules that are used for many essential biochemical processes in the body. They are synthesized by a de nova synthetic pathway and excreted as uric acid. Uric acid is a primary product of xanthine oxidoreductase and a final oxidation product of purine catabolism. Serum uric acid levels elevate in heart failure and cardiovascular diseases, which are related to increased activity of the purine pathway [15]. The understanding of the correlation between the purine pathway and cardiac disease has increased in humans. Atherosclerosis is caused by endothelial injury from reactive oxygen species (ROSs) [16]. ROSs are decreased by the enzyme xanthine oxidase (X.O.), which catalyzes the conversion of hypoxanthine or xanthine into uric acid [17]. According to some studies, the concentrations of specific purine pathway metabolites, such as guanine, hypoxanthine, adenine, xanthine, and uric acid, are significantly elevated in living individuals with acute coronary syndrome (ACS) before percutaneous coronary intervention (PCI) [18,19]. Although correlations between metabolites of the purine pathway and coronary artery disease (CAD) in living individuals are known, there has been limited understanding in postmortem studies. As such, this study investigates the relationship between the levels of metabolites in the purine pathway and coronary artery disease as a cause of death.

## 2. Materials and Methods

### 2.1. Materials

Methanol, chloroform, deuterium oxide (D_2_O), and 3-(trimethylsilyl)-[2, 2, 3, 3-d4]-1-propionate sodium salt (TSP) were purchased from Sigma Aldrich (Saint Louis, MO, USA).

### 2.2. Subjects and Study Design

**Figure 1:**
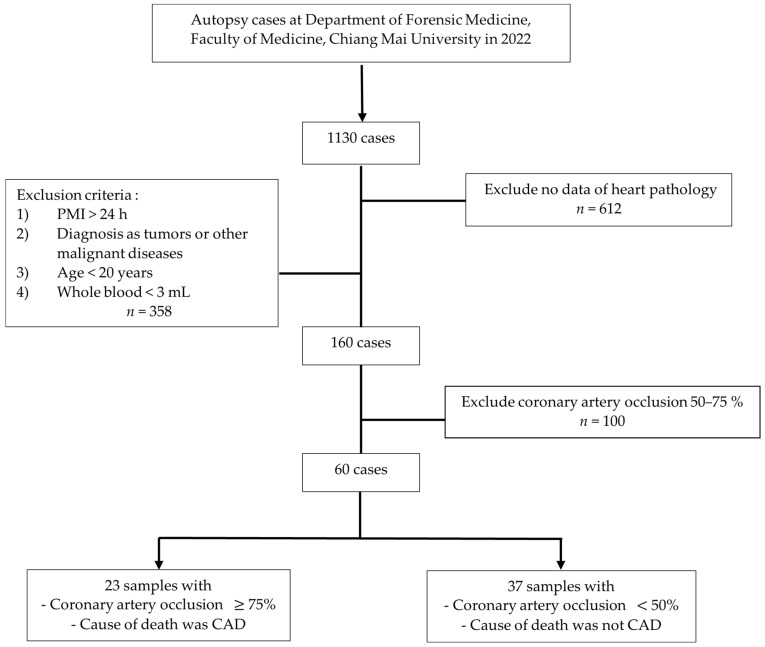
The flowchart of the details of the sample selection process based on the exclusion criteria.

All study cases which underwent autopsy at the Department of Forensic Medicine, Faculty of Medicine, Chiang Mai University, in 2022 were included here. Bodies with a history of tumors and malignancy, a postmortem interval (PMI) of more than 24 h, an age of less than 20 years old, no heart pathological data, and insufficient whole blood samples were excluded. The maximum value of coronary occlusion, which is more than 75 percent, was classified as the CAD group. The processes for sample collection are shown in Figure 1. This study (which has the study code FOR-2562-09155) was approved by the Ethics Committee of the Faculty of Medicine, Chiang Mai University, Thailand.

### 2.3. Collection and Preparation of Blood Specimens

At least 3 mL of heart blood samples were collected in heparin tubes and stored at 80 °C before the analysis. The blood samples were suspended by adding acetonitrile in a ratio of 1:1 and mixed for 10 min. Subsequently, the mixture was centrifuged at 4000 RPM for 10 min. The supernatant was separated and lyophilized. Then, 0.6 mL of 0.1 M TSP was dissolved in D_2_O. The metabolite levels were measured using NMR 500 MHz, employing a technique to suppress water resonance.

### 2.4. Acquisition Parameters

The proton NMR spectrum was recorded and acquired using a Bruker AVANCE 500 MHz instrument (Bruker, Bremen, Germany) equipped with a Carr–Purcell–Meiboom–Gill (CPMG, RD—90°, (t—180°), n—acquire) pulse sequence for the ^1^H-NMR measurements. The spectra were acquired at 27 °C with water suppression pre-saturation. Parameters included 16 scans, a 1 s relaxation decay, a 3.95 s acquisition time, an 8278.146 Hz spectral window, a 0.126 Hz free induction decay (FID) resolution, and a 60.40 dwell time (D.W.). A 90° pulse with 16 signal averages (NSAs) was applied. Baseline and phase correction were carried out by using TopSpin 4.0.7 software. We analyzed spectra from 0 to 12 ppm, normalizing data to the total integrated area. Metabolite resonances were identified using human databases [20]. TSP served as an internal standard, quantifying 24 energy-related metabolites across all samples.

### 2.5. Internal Standard (I.S.)

Trimethylsilyl propanoic acid (TSP) was chosen as the internal standard. TSP is the substance of choice for this application because all 14 protons within TSP shared the same chemical environment. This quality ensures that the signal appears at a singular position at 0 ppm and 500 MHz. In particular, this signal arises from a region with a greater intensity of the magnetic field than that of other protons. In organic compounds, TSP exhibits non-reactivity and has a low boiling point. This being the case, it was convenient to extract from the sample.

### 2.6. Quality Control (Q.C.)

Separate preparations were made for the quality control (Q.C.) samples on the ^1^H-NMR platforms. They were necessary for standardizing the system before, during, and following the analysis in order to observe the system and minimize analytical variation. The quality control samples were prepared by combining and properly mixing an equal quantity of each blood sample. The specimens underwent the same procedure as the samples, according to each of the previous steps. The non-targeted metabolites were investigated by using the methods described.

### 2.7. Peak Assignment and Chemical Identification

Each chemical compound was identified by using the Human Metabolome Database (HMDB), retrieved in March 2023, and a previously published paper (Wishart et al., 2013). The analysis of peak acquisition and J-coupling value was performed using Bruker TopSpin version 4.0.7 software. The interpretation of the NMR spectra relied on the utilization of chemical shift values. These values played a key part in identifying the location of the signal for integration, determining the integrated area beneath the signal, analyzing spin–spin coupling, examining signal patterns, and evaluating the coupling constant. Each peak of every non-targeted metabolite needed to be identified and adjusted by less than 0.01 when compared to the HMDB database.

### 2.8. ^1^H-NMR Data Analysis

MestRenova Software (version 12.0.0, MestreLab Research, Santiago de Compostela, Spain) was utilized to perform data exporting and facilitate spectrum visualization. Furthermore, MetaboAnalyst free online software (version 5.0) (http://www.metaboanalyst.ca/MetaboAnalyst, accessed on 1 June 2023) was employed to identify and portray metabolic pathways.

### 2.9. Statistical Analysis

Data are presented as median. The Kolmogorov–Smirnov test was used to check for normality. The Mann–Whitney U-test was used to compare the means of the two groups. The correlation between metabolite levels and the group was calculated using the Point-Biserial correlation coefficient. A *p*-value of less than 0.05 was considered significant.

## 3. Results

### 3.1. Demographic Data of Participants

Sixty cases were separated into two groups: the CAD group (*n* = 23) and the control group (*n* = 37). The CAD and control groups comprised 20 and 25 males, respectively. Regarding other heart diseases, the category encompasses a range of conditions, including atrial fibrillation, tetralogy of Fallot, dilated cardiomyopathy, pulmonary atresia, and other unspecified heart diseases. Age, gender, and underlying diseases were similar between the groups, and the results are presented in Table 1.

### 3.2. Determination of Metabolites in Blood Samples via ^1^H-NMR

**Figure 2:**
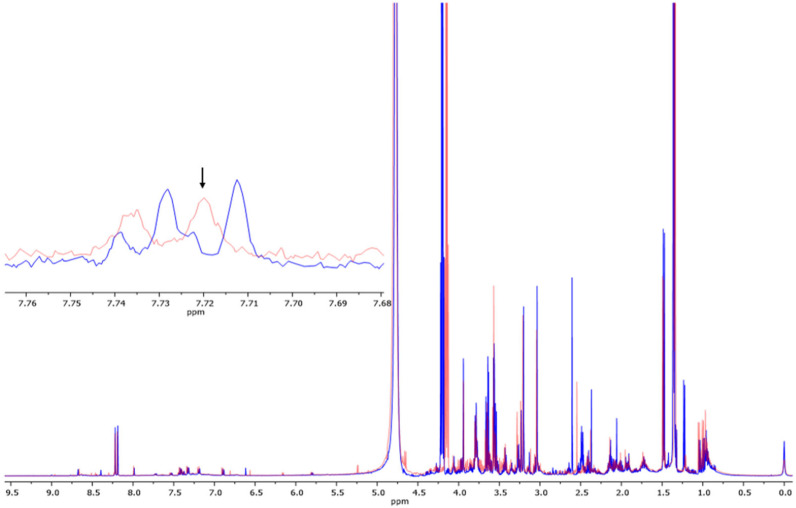
^1^H-NMR chromatogram of the heart blood sample between CAD (blue) and control (red) groups. When TSP = 0.00, L-glutamine = 2.42, deoxyribose 5-phosphate = 3.49, N-acetylneuraminic acid = 3.73, guanosine = 3.92, allantoin = 4.12, GDP = 4.23, inosine monophosphate = 4.35, GMP = 4.37, adenyl succinic acid = 4.58, allantoic acid = 5.34, inosine = 6.09, guanosine diphosphate = 6.10, guanine = 7.59, NAD = 7.60, xanthine = 7.72 (arrow), adenine = 8.00, hypoxanthine = 8.01, adenosine monophosphate = 8.18, adenosine = 8.27, ATP = 8.44, NADPH = 8.45, ADP = 8.58, and GTP = 8.61 ppm.

**Figure 3:**
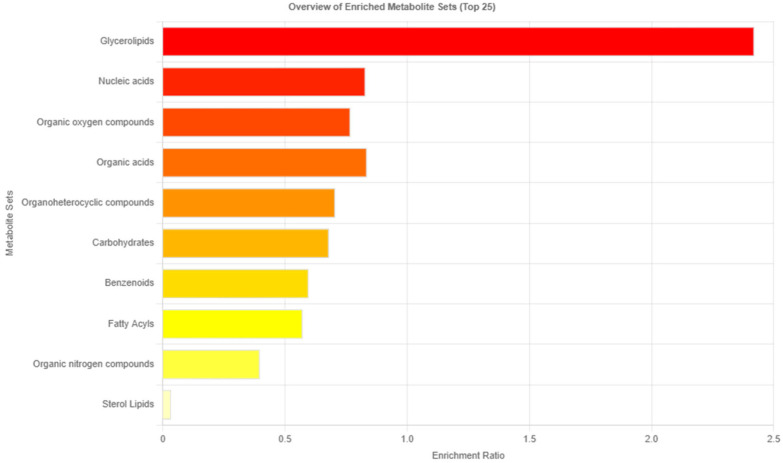
The bar chart illustrates an overview of the non-targeted metabolite groups.

A 500 MHz ^1^H-NMR chromatogram of heart blood samples is illustrated in Figure 2. Metabolites were assigned using the Human Metabolome Database (HMDB, http://www.hmdb.ca/, accessed on 19 February 2023). The chemical compounds were screened in the heart blood samples of the CAD and control groups. They were categorized into glycerolipids, nucleic acids, organic oxygen compounds, organic acids, organoheterocyclic compounds, carbohydrates, benzenoids, fatty acyls, organic nitrogen compounds, and sterol lipids. Nucleic acids, composed of AMP, ADP, ATP, GMP, GMP, and GTP, were involved in the purine pathway. The overview of the group of metabolites is shown in Figure 3.

### 3.3. Analysis of Related Pathways

**Figure 4:**
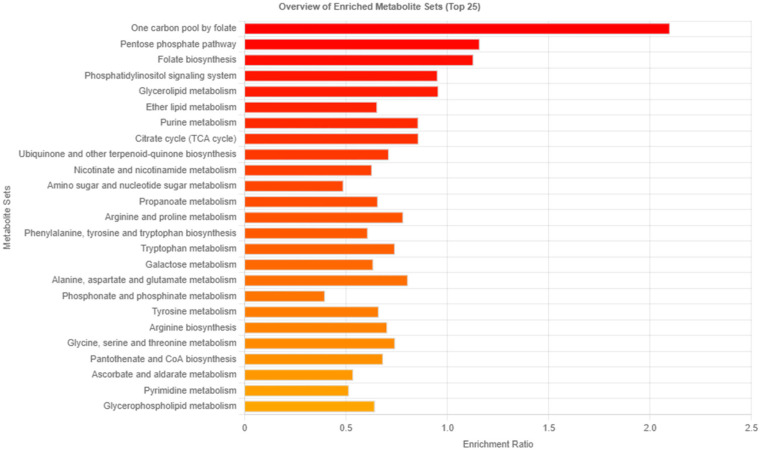
The bar chart represents the enrichment ratio of each metabolite set.

The enrichment ratio for a particular pathway is mapped to metabolites within the pathway based on the KEGG pathway and related to the CAD group. One-carbon pool by the folate pathway, the pentose phosphate pathway, folate biosynthesis, the phosphatidylinositol signaling system, glycerolipid metabolism, purine metabolism, and the citrate cycle presented the enriched ratio of more than 0.8. An overview of the enriched metabolite sets is presented in Figure 4. For the pathway, the folate and pentose phosphate pathways are related to the nutrition of CAD patients. The purine metabolism pathway is related to CAD patients and requires greater description in postmortem cases; therefore, this study focused on only purine metabolism.

### 3.4. Purine Metabolites Analysis in Heart Blood Samples

The 22 metabolites in the purine pathway were found in the CAD and control groups. The levels of metabolites were shown as mean ± S.D. and compared via the Mann–Whitney U-test. The research findings indicated significant alterations in purine metabolites associated with coronary artery disease (CAD). GTP, NAD, and xanthine levels significantly decreased (*p* < 0.05) in the CAD group compared to the control group. Conversely, adenine and deoxyribose 5-phosphate levels significantly increased (*p* < 0.05) in the CAD group. The ratios of guanosine/GMP and ADP/ATP significantly decreased (*p* < 0.05) but that of guanosine/guanosine significantly increased (*p* < 0.05) in the CAD group. All results are presented in Table 2.

### 3.5. Relationship between Metabolites in the Purine Pathway and CAD Group

**Figure 5:**
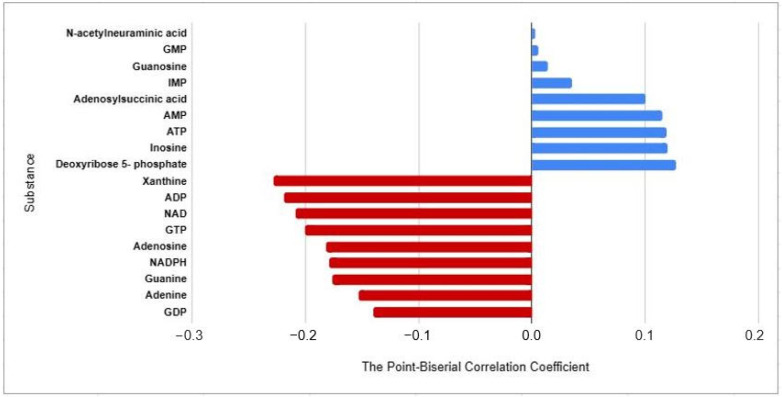
Bar plot presents a correlation of CAD and the purine metabolites by the Point-Biserial correlation coefficient. ADP = adenosine diphosphate, AMP = adenosine monophosphate, ATP = adenosine triphosphate, GDP = guanosine diphosphate, GMP = guanosine monophosphate, GTP = guanosine triphosphate, IMP = inosine monophosphate, NAD = nicotinamide adenine dinucleotide, and NADPH = nicotinamide adenine dinucleotide phosphate hydrogen.

The Point-Biserial correlation coefficient was evaluated for correlations. ADP, GTP, NAD, and xanthine levels showed weakly negative correlations with adenine. Conversely, deoxyribose 5-phosphate and guanosine showed weakly positive correlations within the CAD group. These correlations are shown in Figure 5.

### 3.6. Analysis of Specific Biomarkers for the CAD Group

**Figure 6:**
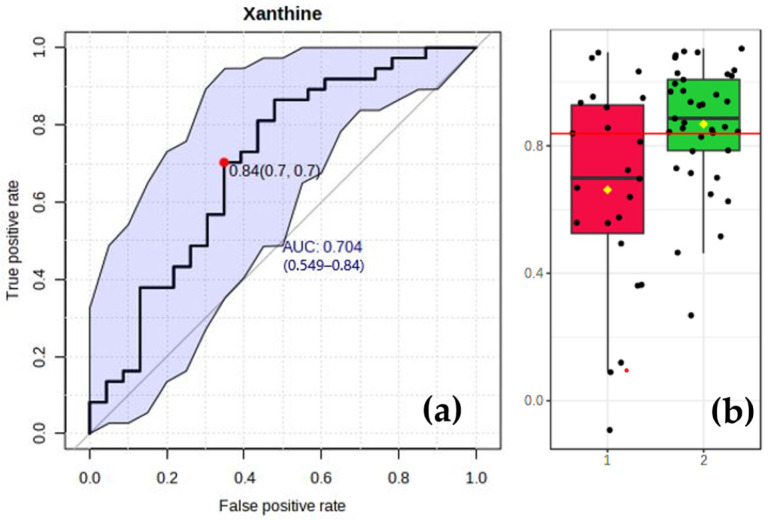
ROC curve analysis (*n* = 60) of the predictive value of xanthine in CAD (**a**). Comparison of xanthine levels in the CAD (*n* = 23, red) and control (*n* = 37, green) groups (**b**). Red line indicates optimum cutoff at 0.84.

Among the metabolites in the purine pathway, xanthine appeared to play a significant role as a predictive metabolite for CAD, with an area under the curve (AUC) value of 0.7 (and a reported confidence interval that excludes 0.5). A receiver operating characteristic (ROC) curve analysis of the predictive value of xanthine in CAD and the boxplot of xanthine level in the two groups are demonstrated in Figure 6.

## 4. Discussion

According to our findings, there are many metabolic pathways related to CAD postmortem cases. Purine was presented as a predominant pathway and correlates most with CAD. Xanthine level is the biomarker with the most potential for postmortem CAD diagnosis.

The samples were divided into two groups according to the percentage of coronary artery occlusion. The percentage of occlusion was categorized by heart pathology because it was more accurate than visual examination. The control group consisted of individuals who died from traffic injury, hanging, neck compression, falling, and gunshot injury, with coronary stenosis less than 50 percent, while the CAD group enrolled more than 75 percent [21,22,23], and the cause of death in the CAD group was coronary occlusion. Heart pathological information in the range of 50 to 75 percent was omitted because this range cannot precisely diagnose CAD as a cause of death. The data distribution exhibited a higher number of males than females due to male autopsy cases having higher incidents than females. The underlying diseases were analyzed, and the results showed that the diseases did not relate to CAD. In the control group, two cases were diagnosed as gout disease and associated with the purine pathway. The purine metabolites were of a normal range except for hypoxanthine and xanthine levels, which were reduced. The limitation of the ^1^H-NMR technique is that uric acid cannot be determined because it stands at the same position as water (4.8 ppm). We therefore hypothesize that uric acid levels might also decrease.

Many non-targeted metabolites were detected in the blood samples via NMR-based metabolomics and categorized into ten groups. Glycolipids presented the highest enrichment ratio in the CAD group. In CAD patients, lipid profiles include phospholipids, triglycerides, glycolipids, glycerophospholipids, and sphingolipids associated with CAD [24,25]. Glycerolipids, specifically triglycerides, are elevated after death compared to antemortem samples [26]. The change after death, especially autolysis and putrefaction, might interfere with blood glycolipid concentration and require further investigation.

One-carbon (1C) metabolism is mediated by folate as a cofactor [27] and controls mitochondrial energy metabolism [28]. It includes purine and thymidine synthesis, amino acid homeostasis, and redox defense. The relationship between the one-carbon pool by folate and folate biosynthesis was not significant in the CAD group. Homocysteine is a component of folate metabolism; however, it has been changed to undergo CAD conditions [29]. The role of the pentose phosphate pathway is significantly related to vascular endothelial maturation [30]. The phosphatidylinositol signaling system encompasses the process of the phosphorylation and dephosphorylation of inositol phospholipids, which can potentially cause CAD [31]. These pathways are still not entirely clear and can be disrupted by postmortem changes. The purine pathway plays a crucial role in cellular processes involved in one-carbon metabolism, the pentose phosphate pathway, energy metabolisms, DNA and RNA synthesis, and energy, as well as cell signaling [32]. Therefore, in this study, we focused on purine metabolism related to CAD.

There is currently no evidence to prove that metabolites of the purine pathway change postmortem. Several physiological and biochemical transformations, including reduced oxygen circulation, modified enzymatic processes, cellular breakdown, and the cessation of the metabolite synthesis process, carry on after death [33]. These factors have an impact on metabolite levels [34,35,36]. Hypoxanthine, an intermediate in purine breakdown, transforms into uric acid through xanthine oxidase. Several studies have reported that hypoxanthine content increases in humans and animals within 24 h of death [37,38]. The outcomes of our study revealed that deoxyribose 5-phosphate, adenine, and guanine/guanosine increased, whereas GTP, NAD, xanthine, the ratio of guanosine/GMP, and ADP/ATP levels significantly decreased. After death, the levels of expression of purine metabolites in the CAD group changed.

Age, obesity, hypertension, smoking, and hypercholesterolemia are risk factors for coronary heart disease and are linked to CHD pathogenesis. Glycolytic disturbances in insulin-resistant and hyperuricemia states relate to CAD [39]. Uric acid production is connected to glycolysis and controlled by insulin. Phosphoribosyl pyrophosphate (PPRP) is produced from ribose-5-phosphate generated from deoxy-5-phosphate and ATP molecules. The PPRP is an essential metabolite precursor in the purine pathway.

Purine nucleotides, including ATP, ADP, AMP, β, γ-methylene-ATP, diadenosine-tetraphosphate, and polyadenylic acid, are essential molecules in the development and progression of CAD. High adenine levels induce coronary endothelium, leading to an increase in coronary blood flow [40]. The levels of ATP and GTP in endocardial cells of CAD patients associated with left ventricular hypertrophy were decreased [41]. ATP and its metabolites showed a decreased tendency after death [42]. These two phenomena might appear in CAD cadavers. Therefore, the ATP level did not change between the groups in this study.

Superoxide, the reactive oxygen species, affects the energy production and perfusion of myocytes. Superoxide is generated by the degradation of hypoxanthine into xanthine and has been discovered in the endothelia of coronary arteries as well as myocytes of patients suffering from myocardial injury [43]. Our study showed that the xanthine level decreased significantly in the CAD group, compared with the control group. The result obtained was opposed to CAD patient studies in that the xanthine level was increased [43]. These might affect enzyme activities that are involved in transforming hypoxanthine into xanthine in postmortem change. The xanthine level of the CAD group is more widely distributed than the control group. It might be impacted by antemortem factors in the CAD group, such as smoking, medication, nutrition, and environmental conditions.

The guanosine/GMP ratio significantly increased, but the guanine/guanosine ratio decreased in the CAD group compared to the control group. Guanosine/GMP and guanine/guanosine reflected 5′-nucleotidase and purine nucleoside phosphorylase activity related to the CAD group. 5′-nucleotidase activity increases with age and inversely relates to heart recovery from ischemia [44]. Purine nucleoside phosphorylase regulates NAD synthesis and requires further investigation. NAD is a vital molecule in cell biology and human physiology. A reduction in NAD levels is associated with CVDs, heart failure, and arrhythmia [45]. The blood NAD level in the CAD group was depleted compared with that of the control group. Compared with the CAD patients, the blood NAD level upon death was also reduced. With confirmation of CAD as a cause of death, xanthine might be confirmed with NAD for prediction, and machine learning needs to be developed.

**Figure 7:**
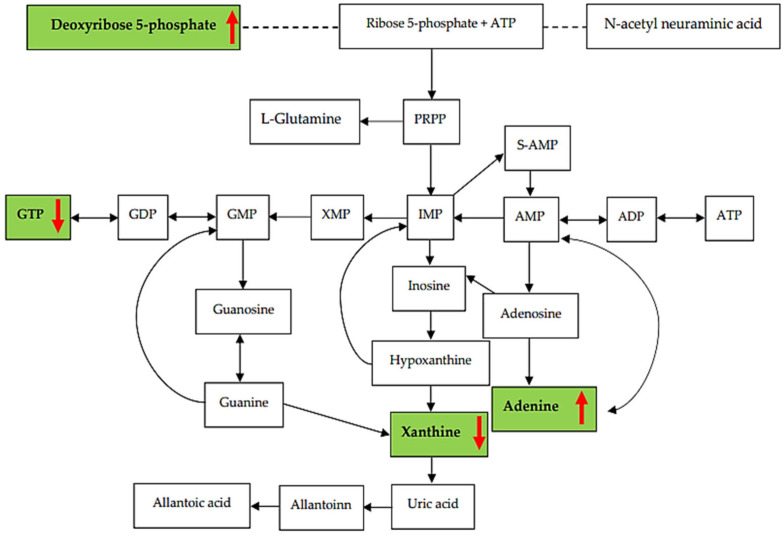
Summary of the purine pathway displayed in the CAD group. Red arrows represent significant increase (↑) and decrease (↓).

Nuclear magnetic resonance spectroscopy (NMR) can be used to detect metabolomic changes in cells, tissues, and body systems [46]. The advantage of the NMR technique is the lower analysis time, easy sample preparation, small sample volume, and non-destructive analysis. It is a sensitive tool for forensic investigations because metabolic changes can be detected, especially in acute myocardial injury [47,48]. These findings might aid in determining the cause of death in cases of SUND. Decomposed organs are the main problem in determining the cause of death. Using metabolite profiles derived from blood or bone might greatly benefit forensic investigations. Uric acid levels should be assessed via liquid chromatography combined with the ^1^H-NMR method. The postmortem interval, the decomposition process, and the underlying disease are potential effects of purine pathway metabolism and should be further studied. As an overview, our findings were that deoxyribose 5-phosphate and adenine significantly increased, whilst GTP and xanthine significantly decreased. The overall purine pathway in CAD is presented in Figure 7.

## 5. Conclusions

Xanthine, GTP, NAD, deoxyribose 5-phosphate, and adenine were found to be related to CAD. The heart blood xanthine level may be developed as a diagnostic biomarker. Postmortem changes and skeletal remains are the key challenges for diagnosing CAD as a cause of death.

## Figures and Tables

**Table 1:** Demographic data of the CAD and control groups.

Factors	CAD (*n* = 23)	Control (*n* = 37)	*p*-Value
**Age (years)**	63 ± 15	56 ± 13	0.063
**Male (%)**	87.0	68.4	0.094
**Hypertension (%)**	52.2	26.3	0.051
**Diabetes mellitus (%)**	21.7	15.8	0.594
**Dyslipidemia (%)**	17.4	13.2	0.685
**Gout (%)**	0	7.9	0.165
**Renal insufficiency (%)**	4.4	5.3	0.856
**Other heart diseases (%)**	21.7	7.9	0.134

The values are presented as mean ± S.D. The variables were compared via the Mann–Whitney U-test.

**Table 2:** Purine metabolites in the CAD and control groups.

Metabolites (μM)	HMDB	CAD Group (*n* = 23)			Control Group (*n* = 37)			*p*-Value
		Q1	Median	Q3	Q1	Median	Q3	
Adenine	0000034	1.56	2.81	13.80	3.58	6.77	23.37	0.042 *
Adenosine	0000050	1.45	3.95	9.53	1.89	8.61	15.37	0.171
Adenyl succinic acid (×$10^{2}$)	0000536	0.26	0.88	2.44	0.29	0.66	2.50	0.613
ADP	0001341	0.47	1.32	6.85	1.27	6.70	15.30	0.077
AMP	0000045	0.47	10.14	38.72	1.30	5.60	15.61	0.405
ATP	0000538	0.32	8.14	31.63	2.02	5.34	12.77	0.405
Deoxyribose 5- phosphate (×$10^{2}$)	0001031	1.13	3.07	8.53	0.41	1.26	3.17	0.046 *
GDP (×$10^{2}$)	0001201	0.13	0.31	1.13	0.18	0.44	1.51	0.882
GMP (×$10^{2}$)	0001379	0.29	0.87	2.20	0.32	0.77	2.44	0.814
GTP	0001273	0.36	0.79	3.24	0.82	3.22	8.37	0.018 *
Guanine	0000132	3.85	9.11	20.09	4.83	13.47	31.32	0.164
Guanosine (×$10^{2}$)	0000133	0.65	2.02	6.95	0.47	0.89	2.29	0.084
Hypoxanthine	0000157	1.93	8.36	40.36	4.11	18.17	38.72	0.319
IMP (×$10^{2}$)	0000175	0.24	0.59	1.75	0.24	0.56	1.83	0.766
Inosine	0000195	0.62	11.87	43.62	1.15	7.49	18.40	0.258
L-glutamine (×$10^{2}$)	0000641	0.60	1.03	2.08	0.50	1.40	5.01	0.847
N-acetylneuraminic acid (×$10^{2}$)	0000230	0.76	1.98	4.11	0.51	1.13	3.09	0.157
NAD	0000902	0.61	1.54	4.29	2.06	3.81	7.03	0.036 *
NADPH	0000221	0.43	1.32	6.85	0.74	4.04	11.71	0.069
Xanthine (×$10^{2}$)	0000292	0.07	0.18	0.44	0.20	0.37	0.74	0.030 *
GDP/GTP (×$10^{2}$)	-	0.08	0.36	1.11	0.05	0.25	0.62	0.251
GMP/GDP	-	1.44	1.93	2.23	1.36	1.60	2.00	0.109
Guanosine/GMP	-	1.14	2.32	5.84	0.72	1.25	2.37	0.026 *
Guanine/guanosine	-	0.01	0.04	0.15	0.06	0.16	0.30	0.003 *
IMP/GMP	-	0.74	0.80	0.91	0.70	0.78	0.90	0.155
IMP/AMP	-	5.94	6.99	77.23	6.05	9.70	48.68	0.538
Inosine/IMP	-	0.05	0.17	0.19	0.02	0.13	0.19	0.425
Hypoxanthine/inosine	-	0.27	2.52	4.63	1.09	4.04	6.54	0.099
Xanthine/hypoxanthine	-	1.12	1.60	5.63	1.27	3.70	7.54	0.277
Xanthine/guanine	-	0.99	2.14	5.78	1.50	2.42	4.62	0.569
AMP/adenyl succinic acid	-	0.01	0.10	0.12	0.02	0.08	0.13	0.744
Adenyl succinic acid/IMP	-	1.16	1.30	1.47	0.96	1.25	1.42	0.233
ADP/ATP	-	0.09	0.38	1.55	0.45	1.35	2.53	0.031 *
Adenine/adenosine	-	0.29	1.24	2.97	0.54	1.62	3.83	0.342
Adenosine/AMP	-	0.20	0.85	1.96	0.53	1.66	3.37	0.127

The values are expressed as the Q1, median, and Q3. * Denotes a statistically significant difference between the groups when using the Mann–Whitney U-test (*p* < 0.05). ADP = adenosine diphosphate, AMP = adenosine monophosphate, ATP = adenosine triphosphate, GDP = guanosine diphosphate, GMP = guanosine monophosphate, GTP = guanosine triphosphate, IMP = inosine monophosphate, NAD = nicotinamide adenine dinucleotide, and NADPH = nicotinamide adenine dinucleotide phosphate hydrogen.

## Data Availability

Data are unavailable due to privacy or ethical restrictions.

## References

[1] GBD 2017 Causes of Death Collaborators (2018). Global, regional, and national age-sex-specific mortality for 282 causes of death in 195 countries and territories, 1980–2017: A systematic analysis for the Global Burden of Disease Study 2017. Lancet.

[2] Lloyd-Jones D., Adams R.J., Brown T.M., Carnethon M., Dai S., De Simone G., Ferguson T.B., Ford E., Furie K., Gillespie C. (2010). Executive summary: Heart disease and stroke statistics—2010 update: A report from the American Heart Association. Circulation.

[3] Tsao C.W., Aday A.W., Almarzooq Z.I., Anderson C.A., Arora P., Avery C.L., Baker-Smith C.M., Beaton A.Z., Boehme A.K., Buxton A.E. (2023). Heart disease and stroke statistics—2023 update: A report from the American Heart Association. Circulation.

[4] Libby P., Theroux P. (2005). Pathophysiology of coronary artery disease. Circulation.

[5] Aggarwal A., Srivastava S., Velmurugan M. (2016). Newer perspectives of coronary artery disease in young. World J. Cardiol..

[6] Udnoon J., Chirachariyavej T., Peonim V. (2009). Sudden unexpected deaths in different age groups at Ramathibodi Hospital, Bangkok, Thailand: A retrospective autopsy study during 2003–2007. Southeast Asian J. Trop. Med. Public Health.

[7] Rao D., Sood D., Pathak P., Dongre S.D. (2014). A cause of Sudden Cardiac Deaths on Autopsy Findings; a Four-Year Report. Emergency.

[8] Basso C., Aguilera B., Banner J., Cohle S., d’Amati G., de Gouveia R.H., di Gioia C., Fabre A., Gallagher P.J., Leone O. (2017). Guidelines for autopsy investigation of sudden cardiac death: 2017 update from the Association for European Cardiovascular Pathology. Virchows Arch..

[9] Al-Adnani M., Scheimberg I. (2006). How can we improve the rate of autopsies among Muslims?. BMJ.

[10] Netto J., Teren A., Burkhardt R., Willenberg A., Beutner F., Henger S., Schuler G., Thiele H., Isermann B., Thiery J. (2022). Biomarkers for Non-Invasive Stratification of Coronary Artery Disease and Prognostic Impact on Long-Term Survival in Patients with Stable Coronary Heart Disease. Nutrients.

[11] Rahimi R., Dahili N.D., Zainun K.A., Kasim N.A.M., Noor S.M. (2018). Post mortem troponin T analysis in sudden death: Is it useful?. Malays. J. Pathol..

[12] Lai P., Nur Shafina M., Mohd Hilmi S., Nur Shazuwani R., Normaizuwana M., Kunasilan S. (2017). Correlation of troponin T levels in the cardiac sudden death cases at hospital kuala lumpur. Int. J. Forensic Sci. Pathol..

[13] Zribi M., Ennouri H., Turki M., Amar W.B., Grati M., Hammami Z., Ayadi F., Maatoug S. (2021). Diagnostic value of high-sensitivity troponin T in postmortem diagnosis of sudden cardiac death. J. Forensic Leg. Med..

[14] Santisukwongchote K., Amornlertwatana Y., Sastraruji T., Jaikang C. (2019). Possible Use of Blood Tryptophan Metabolites as Biomarkers for Coronary Heart Disease in Sudden Unexpected Death. Metabolites.

[15] Bauer J.A., Moffatt-Bruce S.D., Elton T.S., Feldman D. (2008). Purine metabolism in heart failure: Oxidant biology and therapeutic indications. Congest. Heart Fail..

[16] Bredemeier M., Lopes L.M., Eisenreich M.A., Hickmann S., Bongiorno G.K., d’Avila R., Morsch A.L.B., da Silva Stein F., Campos G.G.D. (2018). Xanthine oxidase inhibitors for prevention of cardiovascular events: A systematic review and meta-analysis of randomized controlled trials. BMC Cardiovasc. Disord..

[17] Meneshian A., Bulkley G.B. (2002). The physiology of endothelial xanthine oxidase: From urate catabolism to reperfusion injury to inflammatory signal transduction. Microcirculation.

[18] Dona A.C., Coffey S., Figtree G. (2016). Translational and emerging clinical applications of metabolomics in cardiovascular disease diagnosis and treatment. Eur. J. Prev. Cardiol..

[19] Jung S., Ahn E., Koh S.B., Lee S.H., Hwang G.S. (2021). Purine metabolite-based machine learning models for risk prediction, prognosis, and diagnosis of coronary artery disease. Biomed. Pharmacother..

[20] Dona A.C., Kyriakides M., Scott F., Shephard E.A., Varshavi D., Veselkov K., Everett J.R. (2016). A guide to the identification of metabolites in NMR-based metabonomics/metabolomics experiments. Comput. Struct. Biotechnol. J..

[21] Yang K.M., Lee S.Y., Kim Y.S., Seo J.S., Lee Y.S., Seo J.W. (2008). Guidelines for forensic assessment of natural unexpected cardiovascular death. Basic Appl. Pathol..

[22] DiMaio D., DiMaio V.J. (2001). Forensic Pathology.

[23] Baroldi G., Falzi G., Mariani F. (1979). Sudden coronary death. A postmortem study in 208 selected cases compared to 97 “control” subjects. Am. Heart J..

[24] Liang Q., Liu H., Zhang T., Jiang Y., Zhang A.-H. (2016). Untargeted lipidomics study of coronary artery disease by FUPLC-Q-TOF-MS. Anal. Methods.

[25] Djekic D., Pinto R., Repsilber D., Hyotylainen T., Henein M. (2019). Serum untargeted lipidomic profiling reveals dysfunction of phospholipid metabolism in subclinical coronary artery disease. Vasc. Health Risk Manag..

[26] Girard C., Scarpelli M.P., Tettamanti C., Palmiere C. (2017). Postmortem evaluation of cholesterol, triglyceride, and apolipoprotein levels. Int. J. Legal Med..

[27] Ducker G.S., Rabinowitz J.D. (2017). One-carbon metabolism in health and disease. Cell Metab..

[28] Rosenberger F.A., Moore D., Atanassov I., Moedas M.F., Clemente P., Végvári Á., El Fissi N., Filograna R., Bucher A.-L., Hinze Y. (2021). The one-carbon pool controls mitochondrial energy metabolism via complex I and iron-sulfur clusters. Sci. Adv..

[29] Otsu Y., Ae R., Kuwabara M. (2023). Folate and cardiovascular disease. Hypertens. Res..

[30] Cherepanova O.A., Byzova T.V. (2022). Pentose phosphate pathway drives vascular maturation. Nat. Metab..

[31] Krajnik A., Brazzo J.A., Vaidyanathan K., Das T., Redondo-Muñoz J., Bae Y. (2020). Phosphoinositide signaling and mechanotransduction in cardiovascular biology and disease. Front. Cell Dev. Biol..

[32] Kanehisa M., Furumichi M., Sato Y., Kawashima M., Ishiguro-Watanabe M. (2023). KEGG for taxonomy-based analysis of pathways and genomes. Nucleic Acids Res..

[33] Donaldson A.E., Lamont I.L. (2013). Biochemistry changes that occur after death: Potential markers for determining postmortem interval. PLoS ONE.

[34] Ith M., Bigler P., Scheurer E., Kreis R., Hofmann L., Dirnhofer R., Boesch C. (2002). Observation and identification of metabolites emerging during postmortem decomposition of brain tissue by means of in situ 1H-magnetic resonance spectroscopy. Magn. Reson. Med..

[35] Jawor P., Ząbek A., Wojtowicz W., Król D., Stefaniak T., Młynarz P. (2019). Metabolomic studies as a tool for determining the postmortem interval (PMI) in stillborn calves. BMC Vet. Res..

[36] Pesko B.K., Weidt S., McLaughlin M., Wescott D.J., Torrance H., Burgess K., Burchmore R. (2020). Postmortomics: The Potential of Untargeted Metabolomics to Highlight Markers for Time Since Death. OMICS.

[37] Rognum T.O., Hauge S., Øyasaeter S., Saugstad O.D. (1991). A new biochemical method for estimation of postmortem time. Forensic Sci. Int..

[38] Zhu B.-L., Ishikawa T., Michiue T., Tanaka S., Zhao D., Li D.-R., Quan L., Oritani S., Maeda H. (2007). Differences in postmortem urea nitrogen, creatinine and uric acid levels between blood and pericardial fluid in acute death. Leg. Med..

[39] Leyva F., Wingrove C.S., Godsland I.F., Stevenson J.C. (1998). The glycolytic pathway to coronary heart disease: A hypothesis. Metabolism.

[40] Nees S. (1989). Coronary flow increases induced by adenosine and adenine nucleotides are mediated by the coronary endothelium: A new principle of the regulation of coronary flow. Eur. Heart J..

[41] Swain J.L., Sabina R.L., Peyton R.B., Jones R.N., Wechsler A.S., Holmes E.W. (1982). Derangements in myocardial purine and pyrimidine nucleotide metabolism in patients with coronary artery disease and left ventricular hypertrophy. Proc. Natl. Acad. Sci. USA.

[42] Warangkool C., Churdsak J., Chaturong K. (2022). Changing of ATP and Its Metabolites in Blood Samples for Post Mortem Interval: In Vitro Study. Indian J. Forensic Med. Toxicol..

[43] Baldus S., Mullerleile K., Chumley P., Steven D., Rudolph V., Lund G.K., Staude H.J., Stork A., Koster R., Kahler J. (2006). Inhibition of xanthine oxidase improves myocardial contractility in patients with ischemic cardiomyopathy. Free Radic. Biol. Med..

[44] Grosso M.A., Banerjee A., St Cyr J.A., Rogers K.B., Brown J.M., Clarke D.R., Campbell D.N., Harken A.H. (1992). Cardiac 5′-nucleotidase activity increases with age and inversely relates to recovery from ischemia. J. Thorac. Cardiovasc. Surg..

[45] Xu W., Li L., Zhang L. (2020). NAD+ metabolism as an emerging therapeutic target for cardiovascular diseases associated with sudden cardiac death. Front. Physiol..

[46] Senn T., Hazen S.L., Tang W.H. (2012). Translating metabolomics to cardiovascular biomarkers. Prog. Cardiovasc. Dis..

[47] Surendran A., Atefi N., Zhang H., Aliani M., Ravandi A. (2021). Defining Acute Coronary Syndrome through Metabolomics. Metabolites.

[48] Lewis G.D., Wei R., Liu E., Yang E., Shi X., Martinovic M., Farrell L., Asnani A., Cyrille M., Ramanathan A. (2008). Metabolite profiling of blood from individuals undergoing planned myocardial infarction reveals early markers of myocardial injury. J. Clin. Investig..

